# Enhancing Intersection Efficiency: A Comparative Analysis of Converting Single-Lane Roundabout to Turbo Roundabout

**DOI:** 10.3390/s25227002

**Published:** 2025-11-16

**Authors:** Kristián Čulík, Alica Kalašová, Peter Fabian, Ján Ondruš

**Affiliations:** Department of Road and Urban Transport, University of Žilina, Univerzitná 1, 01026 Žilina, Slovakia; alica.kalasova@uniza.sk (A.K.); peter.fabian@stud.uniza.sk (P.F.); jan.ondrus@uniza.sk (J.O.)

**Keywords:** turbo roundabout, intersection, traffic engineering, safety, traffic flow, microsimulation

## Abstract

This paper evaluates the operational and safety performance of a conventional single-lane roundabout (SLR) and a turbo roundabout (TR) using a mixed-methods approach. Field traffic counts and turning-movement matrices were collected at the studied intersection in Pezinok (Slovakia). The article describes capacity calculations according to national guidelines (TP 100 and TP 102) and the development of a calibrated and validated microsimulation model. Alternative designs—the existing SLR and a proposed TR conversion—were compared under observed morning and afternoon peak-hour conditions. Performance was assessed using standard operational indicators (delay, queue length, degree of saturation) and qualitative consideration of conflict points and weaving behavior. Simulation results for the case study indicate that the TR design reduced average delays and queue lengths and removed internal weaving compared with the SLR for the tested traffic distributions. However, the observed benefits are conditional: they depend on traffic volumes and turning-movement patterns, and may be reduced under very high total flows or when pedestrian and cyclist impacts are significant. Implications for applicability and limitations of TRs are discussed.

## 1. Introduction

Transportation is an integral part of human life. Currently, road transport suffers from various issues related to safety and travel comfort. The most serious problems in road transport are traffic accidents, traffic congestion, and emissions of air pollutants [1,2]. In addition, traffic also causes noise and takes up land [3]. Despite the best efforts to drive green, if the transport network is congested, it leads to congestion and traffic jams [4,5]. The authors suggest that these problems can be addressed through better traffic management, specifically by supporting modern technologies such as the Internet of Things (IoT), fog computing, cloud computing, and data analytics [6]. However, in some cases, it is also necessary to consider infrastructural modifications and changes in traffic organization.

Key elements that significantly affect the capacity of the road network are intersections. Their capacity and saturation level greatly influence vehicle waiting time and queue length, and cause traffic congestion [7]. More than 44% of all car accidents occur at intersections [8]; however, the type of intersection, its structural design, and visibility conditions have a significant impact on the probability of a traffic accident [9,10].

Currently, there is a prevailing trend in converting traditional at-grade intersections (both uncontrolled and signal-controlled) into roundabouts. This trend is particularly evident in Eastern Europe. According to [11,12], a roundabout is defined as a circular intersection where vehicles yield to enter and flow unidirectionally around a central island, creating a continuous flow at a steady pace. This fundamental characteristic has several advantages as well as disadvantages. Many factors influence the occurrence of an accident, such as the reaction time of the driver [13], the technical condition of the vehicle [14,15], the braking distance [16,17] and, of course, the speed. The primary advantage of roundabouts is the reduction in vehicle speed when passing through the roundabout [18,19]. Accident data analysis provides statistical evidence that intersections are among the locations with the highest number of road fatalities [20]. According to several authors, such as [21,22], the number of traffic accidents at roundabouts is often lower than at other at-grade intersections. Analyses show that converting a traditional intersection into a roundabout can reduce the number of fatal accidents by 65% and injury accidents by 40% [23]. Interestingly, in the Slovak Republic, there are statistics on the number of traffic accidents that occurred within roundabouts (Figure 1). It is striking that over the past five years, only one accident at a roundabout resulted in a fatality. Out of the total number of traffic accidents, those occurring at roundabouts account for only 0.67 to 0.74%.

However, it is not only the speed reduction that makes driving through roundabouts safer than other intersections. Figure 2 shows the conflict points in a traditional crossroad intersection and a roundabout. From the figure, it is clear that entry and exit points are reduced by 50%, and crossing points are eliminated [25].

It is important to note that roundabouts may not be entirely suitable for vulnerable road users—pedestrians and cyclists [26,27]. An interesting study [28] analyzed traffic accident records from 90 intersections in Belgium. According to this study, pedestrians and cyclists are more frequently involved in traffic accidents when roundabouts have cycling lanes passing through them. In Slovakia Republic, the Technical Conditions TP085 [29] regulate the cycling infrastructure design. Cyclists can pass through a roundabout in two ways. They may ride in the main traffic space, but only at small roundabouts (up to 40 m in diameter) and never in TRs; in this case, the cycle lane ends 30 m before the roundabout and resumes 15 m after it. Alternatively, cyclists may be guided outside the roundabout, either on a separate cycle lane with crossings or on a completely independent cycle track located at least 5 m away from the road. Regardless of the construction type of the roundabout, intersections usually result in longer routes for pedestrians and cyclists [30].

The advantages and disadvantages of replacing traditional intersections with roundabouts cannot be generalized, as they depend on specific traffic contexts. Sometimes, the transformation of conventional intersections into roundabouts does not lead to improvements in traffic flow characteristics. Especially in cases where one main direction significantly exceeds the others in terms of traffic intensity, a conventional roundabout is not an ideal solution. Studies [31,32] indicate that most roundabouts handle traffic loads with a single lane around the circle. However, in some cases, this capacity is insufficient, necessitating the reconstruction of these intersections into large roundabouts with two lanes around the circle. This often results in a deterioration of road user safety [33,34]. The causes of the deterioration in traffic safety are described in the following paragraphs.

Analysis of some studies shows that under certain circumstances, TRs can overcome this problem, improve operational performance, and create a safer environment [35]. TR is a special type of roundabout featuring two or more spiral-shaped circulating lanes, into which three or more arms of intersecting roads connect. The spiral arrangement of lanes and their physical separation ensure smooth traffic flow through the intersection without the need for lane changes, thereby eliminating weaving movements within the circulatory roadway and reducing conflict points at the exits. The proper functioning of a TR requires that vehicles be distributed into appropriate lanes for their intended directions (destinations) before entering the intersection. Figure 3 shows the typical layout of basic TR. TRs were developed in the Netherlands in the late 1990s by Lambertus Gerrit Hendrik Fortuijn [36]. The first TR was built in Drachten, in northern Netherlands, during this period. TRs are an alternative that addresses the problem of increasing traffic intensity while improving traffic flow and reducing the risk of collisions. The main difference lies in the fixed traffic lanes, which force drivers to choose their direction in advance and prevent dangerous lane crossing within the roundabout.

Thanks to the specific vehicle guidance, the main advantage of TRs is the reduction in the number of conflict points, thereby increasing safety compared to traditional roundabouts [37]. The circulating lanes usually have physical lane separations that ensure safe passage for vehicles in both the inner and outer lanes. These separations are only interrupted at the entrances to the inner circulating lane. This design prevents lane changes within the roundabout, allowing vehicles to pass smoothly through the intersection, eliminating weaving, and consequently enhancing safety and increasing the roundabout’s capacity [38]. Figure 4 illustrates the reduction in conflict points in TRs compared to two-lane roundabouts.

In Slovakia, the Technical Conditions TP 100 regulate the design and capacity calculations of TRs [39]. According to these technical conditions as well as [37,40,41], TRs are categorized as follows:

- 4-armed TR: basic, egg (oval), knee, spiral, rotor.
- 3-armed TR: stretched-knee, star.

TRs also have their drawbacks. If a TR is implemented in place of an existing two-lane roundabout, all road curbs must be demolished, and the dividing islands and public lighting must be rebuilt. Such construction modifications are extremely costly [42]. It is also important to note that TRs contain crossing points, which a typical SLR does not have. The authors of [43] even assert that, in most situations, a two-lane roundabout provides greater capacity than an equivalent TR. However, they do not deny that TRs offer higher operational safety.

The Technical Conditions TP 100 [39] indicate that TRs have higher capacity. It is assumed that a typical SLR is suitable for an average daily traffic volume of 15,000 to 25,000 vehicles per 24 h. A two-lane roundabout is appropriate for a volume of 16,000 to 32,000 vehicles per 24 h, and a TR for a volume of 25,000 to 40,000 vehicles per 24 h.

The Technical Conditions TP 100 also defines specific types of TRs with indicative capacities (PCU = Passenger Car Unit):

- Basic (two-lane) TR with a maximum capacity of 3500 PCU/h.
- Egg (two-lane) TR with a maximum capacity of 2800 PCU/h.
- Knee (two-lane) TR with a maximum capacity of 3500 PCU/h.
- Spiral (three-lane) TR with a maximum capacity of 4000 PCU/h.
- Rotor (three-lane) TR with a maximum capacity of 4500 PCU/h.

Authors in [44] used the Quick-Scan model, originally developed by the Province of South Holland, to assess the capacity of conventional roundabouts and TRs in South Africa. Their results indicated that TRs could achieve 25–35% greater capacity than comparable conventional roundabouts, provided that the total entering flow remains below 3000–3500 passenger cars per hour. General information on the capacity of roundabouts typically refers to the total capacity, rather than the limiting conditions. The research presented in this article focuses on examining the conditions (the routing of traffic flows passing through the intersection) for achieving a capacity of 3500 PCU/hour.

The authors in the source [45] mention, in addition to TRs, other specialized intersections such as flower roundabouts [42], compact-semi-two-lane roundabouts [46], target roundabouts [47]. A target roundabout is dual one-lane roundabout with different outer diameters, located on two levels. All right-hand turners on both roundabouts have their own separate right-turn bypass lanes [48].

All these intersections occupy large areas and have negative environmental impacts. The authors in [49] discuss the geometry of intersections and their impact on exhaust emissions. According to them, there is still no general unified conclusion on which types of intersections have a positive influence on overall emissions. Nevertheless, roundabouts are at the forefront due to their safety advantages compared to signal-controlled intersections [50,51,52], and their potential advantage in reducing pollutant emissions is often emphasized. This assertion is based on the fact that roundabouts do not necessarily require vehicles to stop, reducing the need for acceleration and deceleration [47,53,54,55,56].

Pollutant emissions can be approximately estimated using simulations [57,58,59], which are more commonly used to assess delay times and queue lengths. According to [60,61], there are three types of simulations:

- Microscopic—models each individual vehicle (or agent), capturing detailed behaviors such as car-following, lane changing, and interactions in small time steps.
- Mesoscopic—represents groups of vehicles or tracks individual vehicles loosely, using some aggregated flow characteristics to simplify interactions to a manageable computational level.
- Macroscopic—uses aggregate variables like traffic density, flow, and average speed to model traffic as a continuum, ignoring individual vehicle behaviors.

In this article, microsimulation was used, which simulates individual types of vehicles in the traffic stream. Currently, a large number of commercial microsimulation tools can be used, such as PTV Vissim [62,63], Aimsun [57,64], Paramics [65,66], and Eclipse SUMO [67,68]. Vissim and Aimsun are frequently compared by authors [69,70,71]. The microsimulation model in this article was developed in Aimsun Next, which was selected primarily due to its availability at the authors’ institution and its proven capability for detailed modeling of roundabout operations. The software enables integrated microscopic and mesoscopic simulations within a single environment, supporting the realistic modeling of vehicle interactions and pedestrian effects. Aimsun is widely used in both research and practice, offering reliable tools for calibration, validation, and statistical evaluation of traffic performance indicators. Its advantages include the ability to analyze lane-based parameters such as delay and queue length, which were key outputs in this study. The primary limitation of the software is the model-specific driver behavior algorithms, which may result in variations in results compared to other simulation tools. To minimize this effect, the model was carefully calibrated using field data, and sensitivity checks were conducted to verify the robustness of outcomes. The authors acknowledge that future research will include comparative simulations in PTV Vissim to evaluate cross-software consistency and to validate further the findings presented in this paper.

Additionally, AutoCAD 2023 was used for drawing the design parameters of the intersection. Many authors also use the Civil 3D extension for intersection design [72,73].

To ensure broader representativeness and robustness of the findings, an additional survey was conducted at another TR. This secondary location serves as an example of an existing implementation, analyzed without any proposed modifications. This inclusion of additional case provides further evidence to support the applicability of the results in different traffic situations.

## 2. Materials and Methods

The assessment of the suitability of a TR at a location currently served by SLR is demonstrated in this article through a case study described in the following sections. The traffic survey was conducted in accordance with the applicable legislation. Generally, every intersection must be evaluated according to the technical conditions TP 102 [25], a TR according to TP 100 [39]. A 12 h traffic survey is necessary to perform this evaluation. Such a survey must be conducted on an average working day (i.e., Tuesday, Wednesday, or Thursday). In the previous text, it was mentioned that microsimulation software can be used to assess intersection design alternatives. In Slovakia, microsimulation can be employed for real-world intersection assessments only in certain specific cases (e.g., in the capital city due to specific regulations).

In this article, both the current state and the proposed solution was assessed using capacity calculations as well as microsimulation in the Aimsun software.

### 2.1. Traffic Survey

The case study was conducted in Pezinok, located 18 km northeast of Bratislava, the capital of Slovakia. Pezinok is a district town in the Bratislava region, bordering the districts of Senec, Bratislava, Malacky, and Trnava. The two second-class roads and one third-class road managed by the Bratislava self-governing region lead through the city. This intersection has significant research and experimental potential, as it notably influences traffic in the town and experiences considerable congestion.

The traffic survey was conducted on Thursday, 26 October 2023, from 6:00 a.m. to 6:00 p.m., thus spanning 12 h. Throughout this period, the situation at the intersection was recorded on video from a point that provided a view of all the arms of the roundabout. The evaluation of the traffic survey was performed in 15 min intervals. The designation of the individual entries into the roundabout is shown in Figure 5. Satellite images are obtained from the source [74].

During the 12 h survey, a total of 13,370 vehicles entered the roundabout from 6:00 a.m. to 12:00 p.m. In the second half of the survey, from 12:00 p.m. to 6:00 p.m., 14,096 vehicles passed through the intersection. The total number of recorded vehicles was 27,466, including 58 cyclists, 106 motorcyclists, 24,269 passenger cars, 2482 light trucks, 349 heavy trucks, and 202 buses.

**Figure 7:**
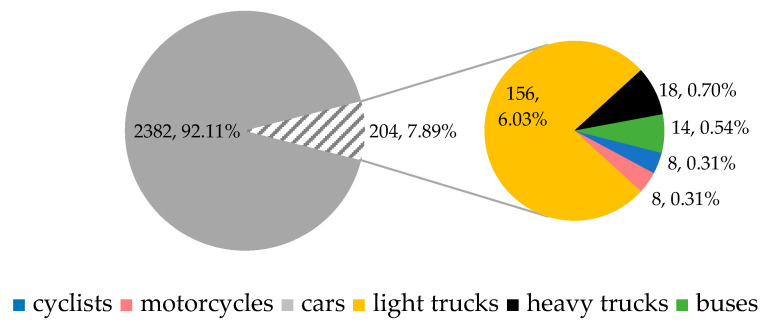
Traffic flow composition during APH. Source: authors.

**Figure 1:**
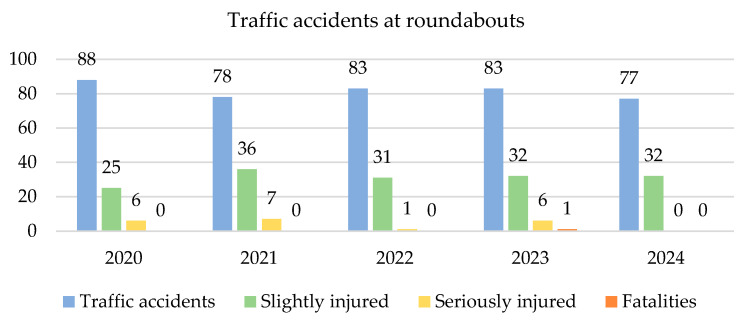
Number of traffic accidents at roundabouts and their consequences. Source: [24].

**Figure 2:**
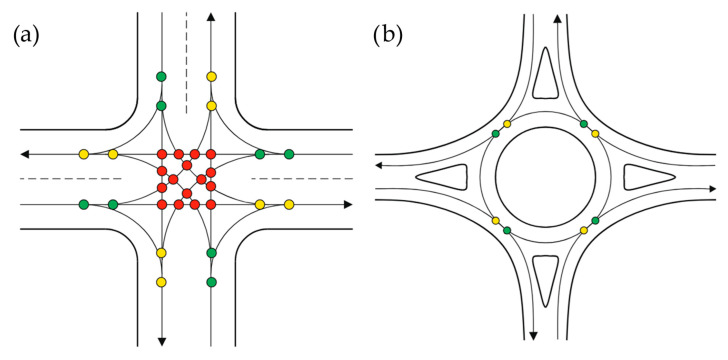
Conflict points: (**a**) at four-lane uncontrolled junction; (**b**) at SLR; yellow—connection points, green—disconnection points, red—crossing points, arrows—driving directions. Source: authors.

**Figure 3:**
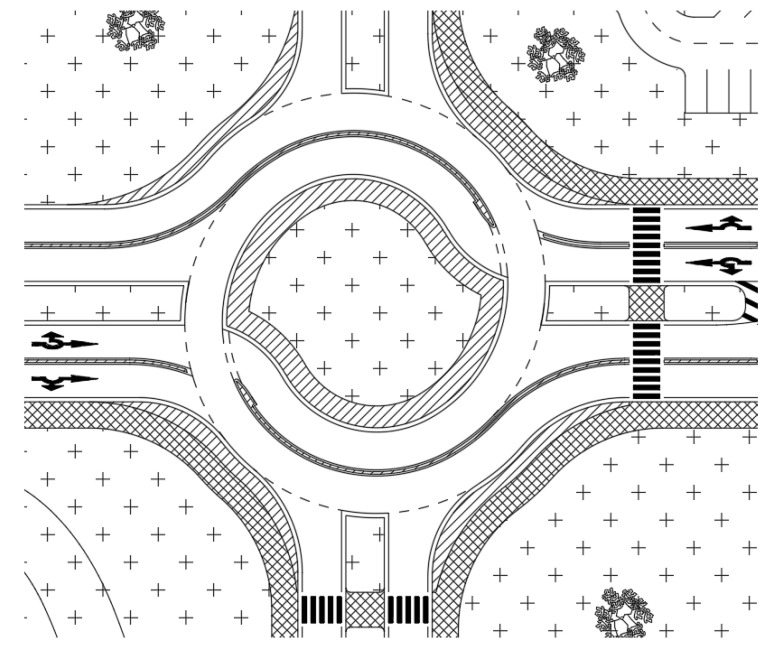
Basic layout of TR. Source: authors.

**Figure 4:**
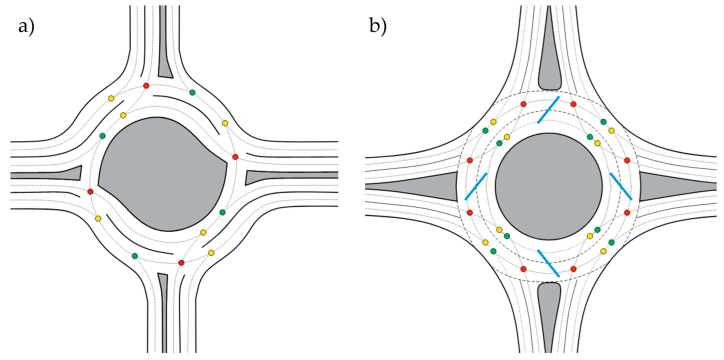
Conflict points: (**a**) at TR; (**b**) at two-lane roundabout; yellow—connection points, green—disconnection points, red—crossing points, blue lines—weaving areas. Source: authors.

**Figure 5:**
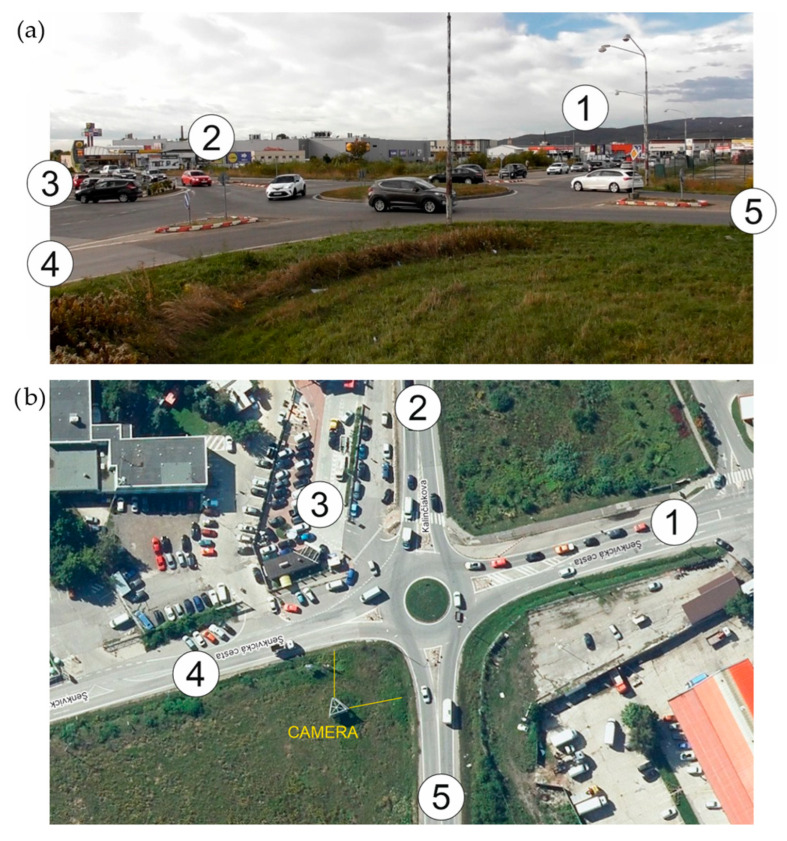
Arms and their numbers of SLR in current state: (**a**) from the camera view; (**b**) from above. Source: authors.

**Figure 6:**
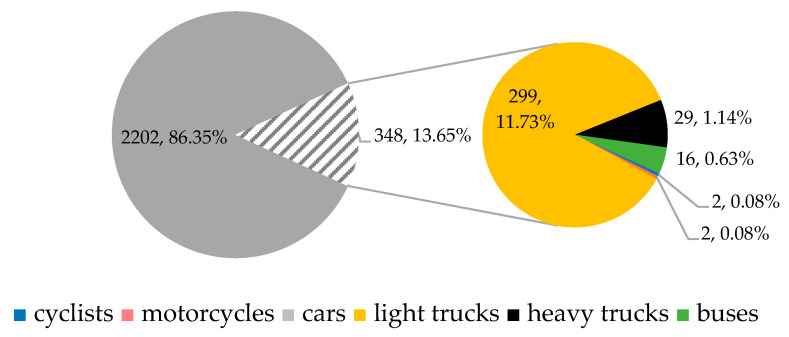
Traffic flow composition during MPH. Source: authors.

For capacity assessment and microsimulation in Aimsun, it is necessary to identify the peak hours separately for the morning and afternoon. The peak hour is defined as the maximum sum of four consecutive 15 min vehicle counts. In this case, the peak hours were identified as follows:

- Morning peak hour (MPH, Figure 6): from 7:30 a.m. to 8:30 a.m., during which 2550 vehicles passed through the roundabout. Of these, 2202 (86.35%) were passenger cars, 299 (11.73%) were light trucks, 29 (1.14%) were heavy trucks, 16 (0.63%) were buses, 2 (0.08%) were motorcycles, and 2 (0.08%) were cyclists.

- Afternoon peak hour (APH, Figure 7): from 3:45 p.m. to 4:45 p.m., during which 2586 vehicles passed through the roundabout. Of these, 2382 (92.11%) were passenger cars, 156 (6.03%) were light trucks, 18 (0.70%) were heavy trucks, 14 (0.54%) were buses, 8 (0.31%) were motorcycles, and 8 (0.31%) were cyclists.

**Figure 8:**
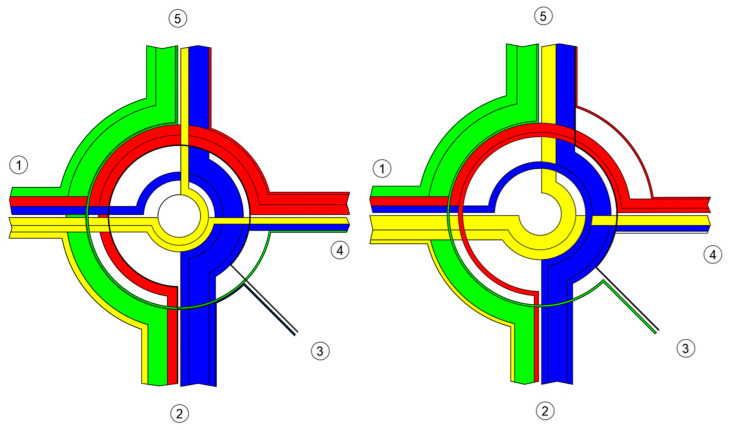
Intersection cartogram during MPH (**left**) and APH (**right**). Source: authors.

The following figure (Figure 8) shows the direction of traffic flows for the MPH (left) and the APH (right).

The total load of the analyzed intersection is in the matrix (Table 1) for MPH and in Table 2 for the APH. Both matrices are in passenger car units (PCU) using the following conversion factors: 0.5 for bicycles, 1.0 for passenger cars, 1.5 for trucks and buses, and 2.5 for heavy trucks.

To reproduce the capacity calculations whose results are presented in this article, in addition to the input matrices, it is also necessary to know the applied growth coefficients described in Section 2.4.

An additional survey was also conducted to complement the analysis of the primary TR. The survey lasted 12 h, from 6:00 a.m. to 6:00 p.m., during which key traffic data were collected. The main focus was on measuring delay times at the roundabout entries, number of vehicles and their direction. Data was gathered using a stationary video camera. The recorded footage was subsequently analyzed to extract vehicle counts and directional movement patterns.

### 2.2. Capacity Assessment

The capacity assessment of the intersection was conducted based on TP 102 [25] for the current state (2023) as well as for the projected states in 2033 and 2043.

The fundamental principle of the capacity calculation primarily involves the calculation of the basic capacity *G_i_* (1), which is influenced by the intensity of the critical traffic flow on the roundabout before the assessed entry *q_k_*, the number of lanes on the circular roadway *n_k_*, and also the time gaps:

- The critical time gap *t_g_* (s) is necessary for one vehicle to enter the roundabout.
- The average follow-up time gap *t_f_* (s) is the time gap between vehicles entering the roundabout, which enter the circular roadway during the one-time gap between two vehicles on the circular roadway.
- The minimum time gap *t_min_* (s) is between vehicles on the circular roadway.

(1) $$G_{i}={\left(1-\frac{t_{min\;}*{\;q}_{k}}{3600\;*\;n_{k}}\right)}^{n_{k}}*\;\frac{3600}{t_{f}}*e^{-\frac{q_{k}}{3600}\;*\;\left(t_{g}-\frac{t_{f}}{2}-t_{min}\right)}$$

The capacity of the entry lane *C_i_* then results from the basic capacity considering the influence of pedestrians through the coefficient *f_f_*_1_. In the case of the analyzed intersection, the influence of pedestrians was considered only on one approach (approach 2) since the other approaches of the intersection do not have pedestrian crossings. The determination of pedestrian influence is based on their intensity at the crossing and the traffic flow intensity on the circulatory roadway. The detailed procedure is described in [25]. Subsequently, the capacity can be calculated using the simple Formula (2):

(2) $$C_{i}=G_{i}*f_{f1}$$

The degree of saturation *g_i_* is given by a simple ratio (3) and indicates how the capacity of the entry lane *C_i_* is utilized by intensity *q_i_*.

(3) $$g_{i}=\frac{q_{i}}{C_{i}}$$

After performing these calculations, all inputs are ready for the determination of the average delay time *w_i_*. The criterion for evaluating the level of service (LOS) at roundabouts is the average waiting time *w_i_* (4) determined for each entry lane.

(4) $$w_{i}=\frac{3600}{C_{i}}+900\;*\;\left(\left(g_{i}-1\right)+\sqrt{{\left(g_{i}-1\right)}^{2}+\frac{8\;*\;g_{i}}{C_{i}}}\right)$$

Subsequently, it is assessed whether, at the design traffic intensity *q_i_* on the assessed entry lane into the roundabout, the value of the average waiting time *w_i_* does not exceed the threshold that defines the required level of service (LOS). The individual levels are presented in Table 3.

A significant parameter is the queue length denoted as *N*_95,*i*_. This is an important parameter that can be used, for example, in the design of entry lane lengths before a roundabout. This parameter indicates that for 95% of the time, the queue at the given entry will be shorter than the specified distance in meters. It is calculated according to Formula (5).

(5) $$N_{95,i}=\frac{3\;*\;C_{i}}{2}\;*\;\left(\left(g_{i}-1\right)+\sqrt{{\left(g_{i}-1\right)}^{2}+\frac{8\;*\;g_{i}}{C_{i}}*\left[-ln\left(0.05\right)\right]}\right)$$

In some cases, it is the duty of the traffic engineer to calculate and assess the capacity of the exit as well. This assessment is required if the pedestrian intensity at the exit crosswalk exceeds 250 pedestrians per hour. It is also necessary to conduct an exit capacity assessment if the intensity of vehicles leaving the roundabout via the given exit, combined with the number of pedestrians crossing, exceeds 1000 equivalent vehicles plus pedestrians.

### 2.3. Microsimulation

**Figure 9:**
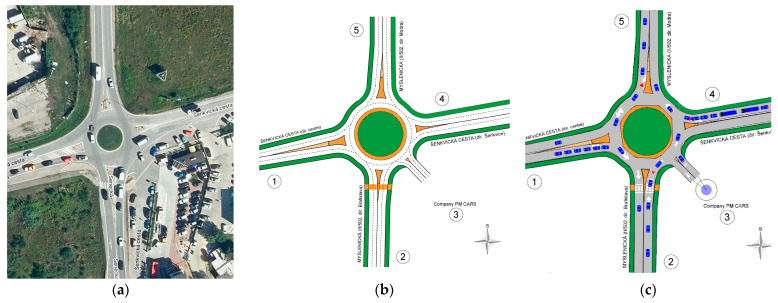
Described intersection: (**a**) satellite image; (**b**) intersection drawing; (**c**) microsimulation. Source: authors.

As a supplement to the capacity assessment, microsimulation was also performed for this roundabout. For the current state, it was necessary to first model the existing traffic network. In the following figure, it can be seen that in the real intersection (Figure 9a), a vector traffic design (Figure 9b), on which it was possible to accurately measure elements affecting the capacity calculation. This base was subsequently used in the microsimulation (Figure 9c).

Calibration and validation of the microsimulation model are the most critical and challenging stages of the model development process. These processes ensure that the model accurately reproduces observed traffic conditions, allowing it to be used for predictive analyses. Calibration typically involves adjusting behavioral and performance parameters to minimize discrepancies between simulated and observed data [75,76]. Validation subsequently confirms that the calibrated model performs consistently under varying traffic scenarios [77].

According to established methodologies, calibration accuracy is commonly evaluated using quantitative indicators such as the GEH statistic, Root Mean Square Error (RMSE), Mean Absolute Percentage Error (MAPE), or Theil’s inequality coefficient [75,77,78]. Acceptable levels of calibration are generally achieved when at least 85% of the simulated traffic volumes result in GEH values below 5, as recommended by the Federal Highway Administration (FHWA) and other recognized guidelines [77,78].

In this study, calibration and validation were conducted in accordance with these internationally accepted procedures, with emphasis on key performance indicators such as delay time and maximum queue length, which correspond to the parameters used in national capacity assessment methodologies. The modeling framework adhered to the recommendations outlined in the Aimsun Next User’s Manual (2023) [79], ensuring consistency with best practices for microsimulation-based analyses.

**Figure 10:**
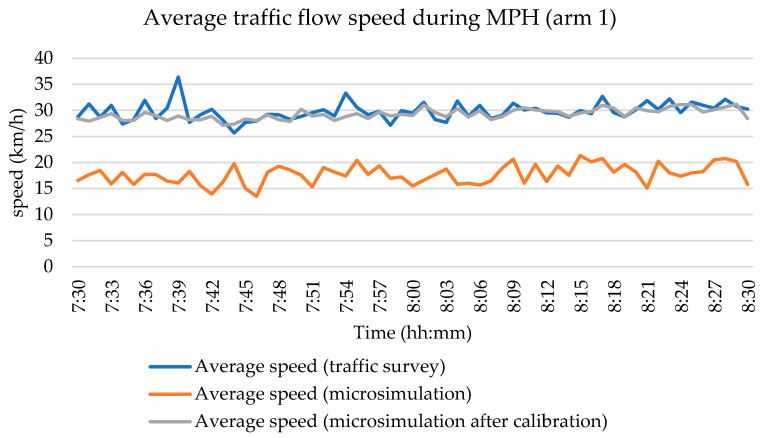
Calibration of the simulation model—speed distribution during MPH. Source: authors.

The calibration and validation of the simulation model were conducted as part of the research process. During the traffic survey of the studied intersection, the Sierzega SR4 device and the ASD radar were employed on arms 1 and 5. The radar device was used to measure vehicle speeds; however, on arm 5, the collected data were considerably distorted, as the traffic flow was practically stationary during the peak hour. For this reason, only the vehicle speeds recorded on arm 1 were considered for calibration purposes (Figure 10). These measured speeds were subsequently used to adjust the simulation parameters, ensuring realistic traffic behavior within the model.

The validation of the simulation was conducted based on an independent parameter, namely the queue length observed at the intersection entries. Not all entries were considered in the validation process; the comparison between observed and simulated queue lengths was performed only for entries 1, 2, and 4, where the most reliable empirical data were available. This approach enabled a more accurate verification of the model’s performance under real-world traffic conditions. Outputs from the validation process are in Table 4.

As can be seen from Table 4, the deviation target set at the beginning of the calibration process (10%) was successfully achieved.

The parameter settings established through calibration and validation were subsequently applied in simulating the TR scenario. The route choice of vehicles within this model was determined according to the fastest available route, with decision-making processes updated at one-minute intervals to reflect dynamic changes in traffic conditions.

In the case of capacity calculations, the primary outputs are the waiting times wi at the individual arms of the roundabout and the queue length *N*_95,*i*_.

The outputs of microsimulation are different metrics. In this study, the following were primarily recorded:

- Delay time (s);
- Maximum queue length (vehicles);
- Number of stops (stops/vehicle);
- Delay time (s).

These values are not entirely comparable to the capacity calculation results since they are obtained through measurements during the simulation and are derived as averages over any number of simulation cycles. However, they provide a new perspective on assessing the quality of traffic at a given intersection. Microsimulations are particularly useful for large roundabouts and turbo roundabouts (TR) [80,81].

**Figure 11:**
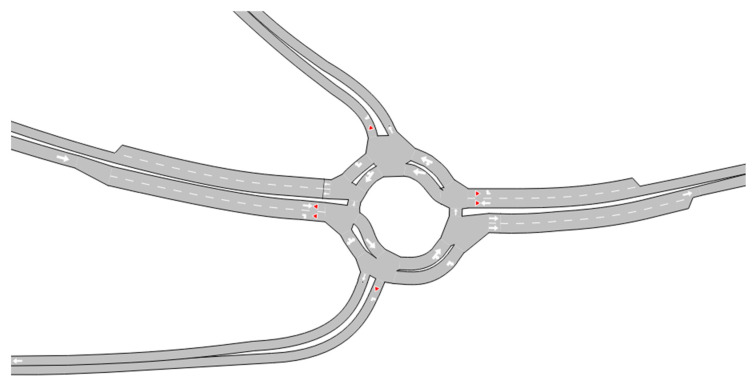
Modeled infrastructure of additional TR in Aimsun.

The additional TR was modeled in Aimsun to reflect its current design and operational characteristics, adhering to the latest regulations, including speed limits. The simulation used data collected during a 12 h survey, specifically vehicle delay times, counts, directional flows, and speeds. The goal of the simulation was to evaluate the operational performance of this intersection and to provide additional support for versatility and applicability of TRs as a universal solution for traffic management. Figure 11 depicts the modeled infrastructure in Aimsun, highlighting the key elements of intersection’s current layout.

### 2.4. Calculation of the Future Intensity

It is quite complex to predict the future load on the road network. In the Slovak Republic, however, the technical conditions TP 070 Prognostication of Future Traffic Volumes on the Road Network up to 2040 [82] are in place. These conditions provide traffic growth coefficients for individual regions, categorized by vehicle type and road category. Separate growth coefficients are provided for light vehicles (in this study, this category includes motorcycles, passenger cars, and cyclists) and heavy vehicles (trucks, heavy trucks, and buses). Considering the intersecting road categories, coefficients for 2nd and 3rd-class roads were chosen. The resulting calculations related to the current period (the year 2023) are shown in Table 5.

Coefficients from Table 5 were used to recalculate new matrices of PCUs for MPH and APH. Since the roundabout involved two roads of various categories, the following procedure was established: For entries from road II/202 (for vehicles from these entries), the coefficient for 2nd class was used, and for entries from Šenkvická road, the coefficients for 3rd class were used.

### 2.5. Hypotheses and Research Questions

All three hypotheses tested in this article relate to solving a practical traffic engineering problem. As mentioned earlier, the hypotheses were tested not only by capacity calculations but also by microsimulation techniques.

**Hypothesis** **H1:** *The TR can reduce waiting time/delay by more than 50% on all arms compared to a SLR.*

**Hypothesis** **H2:** *The microsimulation of a roundabout provides more positive results than capacity calculations according to technical conditions.*

**Hypothesis** **H3:** *The queue length expressed in vehicles is reduced by the TR, but not on all entries compared to the SLR.*

All three hypotheses are evaluated at the end of the next chapter, supported not only by calculations but also by graphical comparisons. This evaluation aims to better understand the differences between capacity calculations and microsimulation.

## 3. Results

When addressing any intersection, it is essential to thoroughly analyze the current state. This article does not provide a detailed calculation of all evaluated parameters, as it suffices to present the most influential operational characteristics of the intersection, such as waiting time, quality of service, and queue lengths.

The following lines describe the outcomes of the analysis and proposal in detail. The procedure for this research task was as follows:

- First, a capacity calculation was conducted according to technical conditions for the current state of the roundabout. This calculation was performed separately for the MPH and APH.
- In the second step, the capacity of the roundabout, specifically the average waiting time, was also determined for future conditions, meaning the vehicle matrix was projected up to the year 2043.
- The third step involved designing a TR suitable for the spatial arrangement in the given area.
- The proposed TR was then assessed for capacity both for the current state and for future traffic intensities.
- In addition to the capacity calculation, simulations were conducted using the microsimulation software Aimsun Next 22.0.1.0.
- The results of the capacity assessment and microsimulation were compared, providing an indication of the findings and allowing conclusions to be drawn from the results.

### 3.1. Capacity Assessment of SLR

The original small roundabout had 5 arms with an outer diameter of 43 m and a single lane on the circular roadway with a width of 6 m. Each arm of the roundabout had one lane both entering and exiting, with the following entries:

- Arm 1: Entry from the city center of Penzinok (Šenkvická road);
- Arm 2: Entry from the capital city via road II/502 (Myslenická street);
- Arm 3: Entry to the p.m. Cars company;
- Arm 4: Entry from the town of Modra via road II/502 (Myslenická street);
- Arm 5: Entry from the village of Šenkvice (Šenkvická road).

**Figure 12:**
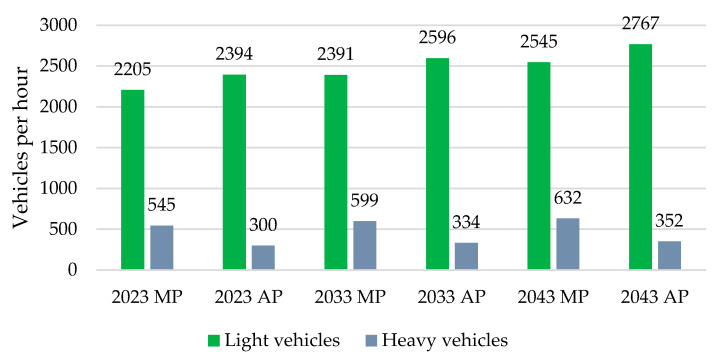
Composition of vehicles during MPH and during APH. Source: authors.

The capacity assessment of the current intersection required establishing traffic flow matrices. The following figure (Figure 12) illustrates the changing overall traffic load on the intersection due to forecasted coefficients. The graph also shows the proportion of light vehicles (cars, vans, bicycles, motorcycles) and heavy vehicles (light trucks, heavy trucks, and buses) contributing to the traffic intensity.

As seen in Figure 12, both peak hours experienced equal numbers of vehicles. However, during the MPH, the intersection had to manage a higher load of heavy vehicles. Additionally, there were changes in the direction of traffic flow, which were depicted using cartograms.

The results of the capacity assessment itself can be found in Table 6. The table includes the average waiting time *w_i_* and the level of service. Given that for arms 4 and 5, the capacity was exceeded during MPH, these two entries were rated as the level of service “F”, resulting in the entire intersection being deemed unsatisfactory.

Regarding APH, the situation at the current SLR intersection did not significantly improve; instead, the load shifted to other arms. During APH, the capacity was exceeded on arm 2, while the other arms had a level of service “D”.

According to the technical conditions, grade “F” is defined as follows [25]: The number of vehicles arriving at the intersection per unit time exceeds the entrance capacity for an extended period. Long queues of vehicles form, causing intolerable waiting times and resulting in the intersection being overloaded.

**Figure 13:**
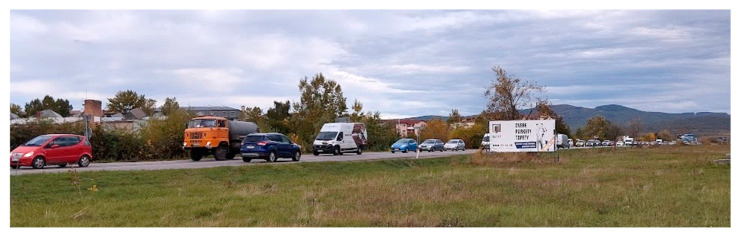
Traffic jam on arm 3 during MPH. Source: authors.

An additional indicator confirming the unsatisfactory situation is the *N*_95_ queue length. This parameter was assessed during MPH with the lengths of the queues at individual arms being 53.1 m (Arm 1), 177.4 m (Arm 2), 3.7 m (Arm 3), 176.5 m (Arm 4), and 312.4 m (Arm 5). An extremely long vehicle queue observed during the traffic survey is captured in the photograph in Figure 13. During the second peak hour, the queue lengths at individual arms were 99.9 m (Arm 1), 295.1 m (Arm 2), 8.7 m (Arm 3), 56.0 m (Arm 4), and 118.2 m (Arm 5).

### 3.2. Proposal of TR

From the previous analysis, it clearly emerges the need to change the current intersection to a different type. Consideration is given to a TR or a two-lane roundabout. In the case of a two-lane roundabout, it would be necessary to convert all entrances to multi-lane, which would not be feasible in this scenario. Therefore, this study focuses on a TR, with the proposal to double lanes on both entry and exit on arms 2 and 5, located on the second-class road.

**Figure 14:**
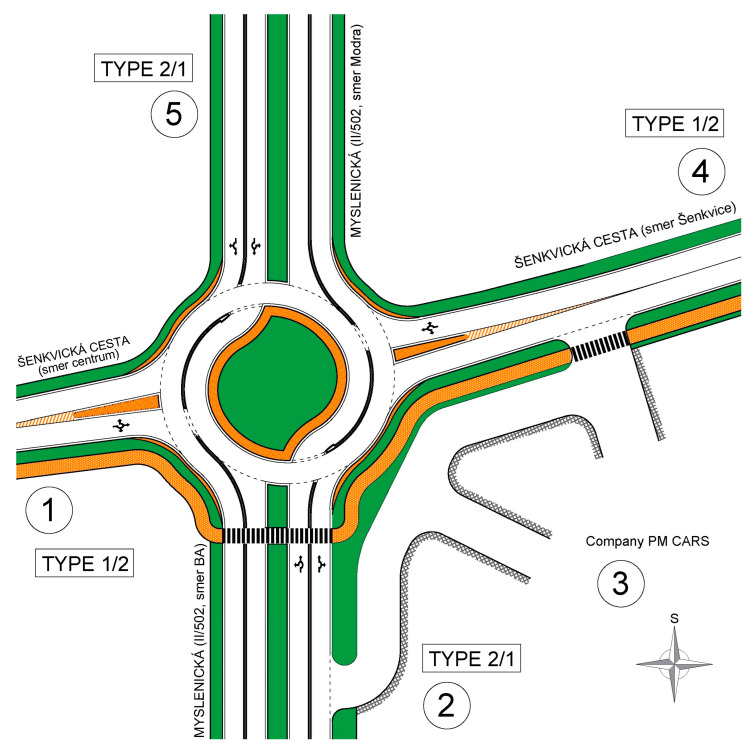
Traffic design of the intersection as input of the microsimulation model; orange – paved areas and sidewalks, green – grassy areas. Source: authors.

The traffic design of the TR can be seen in Figure 14. Entrance 3 is diverted outside the TR because it only carries light traffic to adjacent businesses. If integration of this entrance directly into the roundabout was required, it could be achieved through a single-lane entry between arms 2 and 4.

The individual design elements of the TR correspond to the basic version of the TR, with this design featuring only entry types 1/2 and 2/1. Entry 1/2 means that the entry has one lane, allowing drivers to choose between two lanes within the roundabout. Entry 2/1 indicates that vehicles approach the intersection in two lanes at the entry, with vehicles in the left lane having access to a turbo lane.

### 3.3. Capacity Assessment of Proposed TR

The computational evaluation of this TR proposal was processed according to the technical conditions TP 100, which specify the capacity calculation for all TRs in the Slovak Republic. The capacity calculation did not consider entry no. 3 because it was diverted outside the intersection. However, vehicles entering through this entry were included in entry no. 2.

The results of the capacity calculation are summarized in Table 7. The capacity calculation provides the average waiting time *w_i_* for each lane, thus entries 2 and 5 are divided into left (L) and right (R) lanes.

From the capacity assessment, it is evident that the TR is satisfactory during MPH, with the worst traffic quality grade “D” observed on arm 4 with a waiting time of 32.1 s. In the outlook scenario, this waiting time could stretch up to 73.7 s, which is unfavorable, but the capacity will not be exceeded.

Regarding APH, the results were less positive. For the current state, the traffic quality grade is “E” with a waiting time of up to 53.6 s. According to TP 100, this grade is defined as: “A queue forms that does not diminish under existing load, resulting in very high waiting times. There is a sensitive dependency—small changes in load cause sharp increases in time losses.” When recalculated for the outlook scenario (13.8% to 16.6% higher intensity), even the proposed TR is considered unsatisfactory.

### 3.4. Results of Microsimulations

An additional method to complement the capacity assessment of the intersection is microsimulation. According to [83], a microscopic traffic simulation is a useful tool in analyzing traffic and estimating the capacity and level of service of road networks. Several authors have validated the performance of roundabouts using microsimulation in Aimsun Next 22.0.1.0 software [69,84,85].

**Figure 15:**
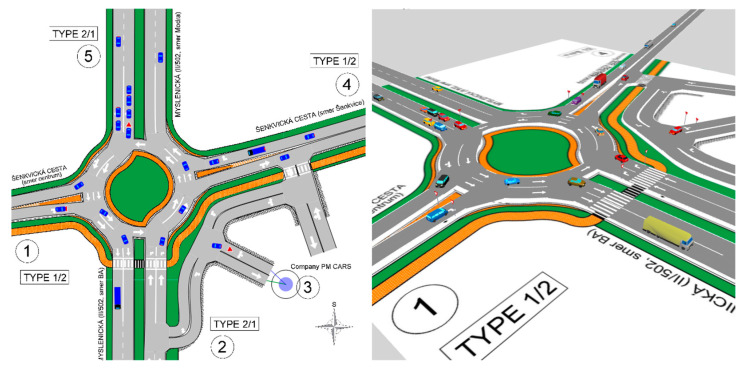
The microsimulation in 2D and 3D view. Source: authors.

In this case, the entire network was modeled based on a vector CAD drawing. Figure 15 shows both the modeled network and the visualization of the ongoing microsimulation in Aimsun.

Compared to capacity calculations, microsimulation also considers the entire bypass route from arm 3. The adequate capacity calculations would require the evaluation of two three-arm intersections and TR. These detailed and separated calculations are not the aim of this study. From the figure, it is clear that the microsimulation accounted for multiple, specifically three, categories of vehicles:

- Category “car,” which included all passenger cars, vans, and motorcycles.
- Category “truck,” which included all trucks weighing over 3.5 tons and tractors.
- Category “bus,” which included all buses over 3.5 tons total weight.

For each simulated scenario, 64 simulations were created, and the average of the recorded characteristics was subsequently determined. Each simulation (replication) lasted one hour with a 10 min “warm-up” of the traffic. The current state of the roundabout and the TR design operated with 5 zones. The source-destination traffic relationships were defined by three matrices depending on the vehicle category. Cyclists (2 per hour) and pedestrians (14 per hour) were neglected in the simulation.

The output of the microsimulation differs from the capacity calculation. For comparison, the following important output parameters were statistically evaluated:

- Delay time (s);
- Stop time (s);
- Max queue (veh).

The selected performance parameters were recorded in microsimulation software using subpaths. Among these, the delay time is the most used output parameter, as it can be associated with the waiting time *w_i_* obtained from capacity calculations. The waiting time represents the total lost time of a vehicle after subtracting the time losses caused by deceleration and acceleration at the intersection. The stop time was analyzed as an additional, informative indicator since capacity calculations do not directly provide it. This parameter expresses the total duration during which a vehicle maintained zero speed due to delay at the simulated intersection. The third parameter, maximum queue length, was included to allow a comparative assessment with the N95 value derived from the analytical capacity model. In the latter case, the parameter represents the queue length that will not be exceeded during 95% of the analyzed time interval.

Table 8 presents the evaluation of SLR and TR from 64 simulations during the MP, while Table 9 shows the results for the AP. Similarly to the capacity calculation, the busiest entries of SLR appear to be arms 4 and 5, where the delay time exceeds 3 and 4 min, respectively. During APH, significant delays were no longer observed. In these results, the capacity calculation and microsimulation diverge considerably. The result values of TR show a reduction in delay time from 77.20% to 98.74% during MPH and a decrease from 82.50% to 95.20% during APH.

**Figure 16:**
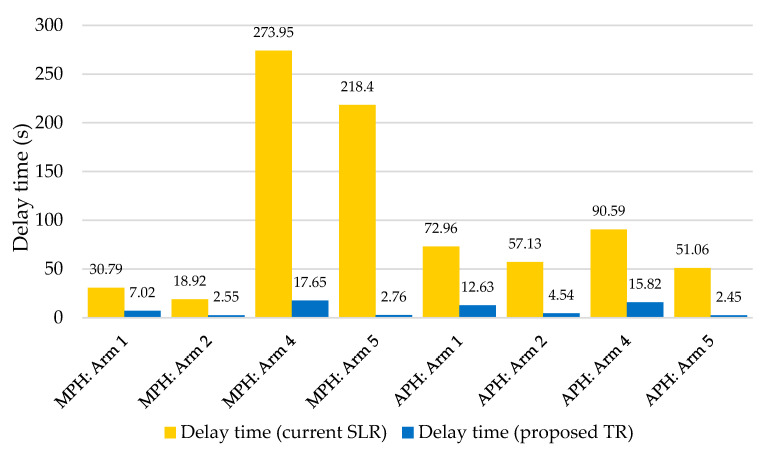
Delay time reduction according to microsimulation—TR compared to SLR. Source: authors.

Previous tables do not include arm 3 (in proposed TR), as this was rerouted outside the intersection. The simplification of the intersection model, specifically the reduction in arm 3, was performed due to its low traffic volumes. Despite this adjustment, the traffic flows associated with this arm were still included in the microsimulation model, ensuring that their influence on the overall traffic dynamics was preserved. In the capacity analysis, these volumes were proportionally redistributed among the remaining arms to maintain the representativeness of the total demand. In practical applications, such conditions are addressed by introducing an auxiliary entry lane connecting to the outer circulatory lane of the turbo roundabout. The following figure (Figure 16) compares the achieved delay time at individual arms of the TR, with the reduction in delay time compared to the original SLR indicated in orange.

### 3.5. Results of the Case Study

As seen from the capacity assessment results, the original SLR is inadequate in the current state (level F for both peak hours). Due to the formation of extensive queues (maximum N95 according to the calculation of 312.4 m), this intersection was selected for evaluation and traffic proposals.

During this research task, the following hypotheses were verified:

**H1:**  *The TR can reduce waiting time/delay by more than 50% on all arms compared to a SLR.*

Assessment to H1: It is not entirely straightforward to compare waiting times between the original SLR and the TR, as the number of entries and lanes changed in the proposal, with the TR having at least two dual-lane entries and exits. Therefore, entry 3 is not included in the following comparison, and in the case of multi-lane entries, the worse value is taken for comparison. The reduction in average waiting time *w_i_* can be evaluated as follows:

- MP: Arm 1: 22.1 s → 16.0 s (27.6% reduction).
- MP: Arm 2: 63.6 s → 7.1 s (88.8% reduction).
- MP: Arm 4: 119.3 s → 32.1 s (73.1% reduction).
- MP: Arm 5: 206.8 s → 11.2 s (94.6% reduction).
- AP: Arm 1: 34.6 s → 11.8 s (65.9% reduction).
- AP: Arm 2: 187.2 s → 11.7 s (93.8% reduction).
- AP: Arm 4: 33.4 s → 53.6 s (60.5% increase).
- AP: Arm 5: 37.3 s → 7.6 s (79.6% reduction).

Although the TR ensures a reduction in waiting time, this is not always the case. At arm 4, there was an increase in waiting time of 60.5%. The same comparison can be made with simulations of the original SLR and the new TR. In this case, it is the delay time parameter again excluding arm 3, which is routed outside the proposed TR:

- MP: Arm 1: 30.79 s → 7.02 s (77.2% reduction).
- MP: Arm 2: 18.92 s → 2.55 s (86.5% reduction).
- MP: Arm 4: 273.95 s → 17.65 s (93.6% reduction).
- MP: Arm 5: 218.40 s → 2.76 s (98.7% reduction).
- AP: Arm 1: 72.96 s → 12.63 s (82.7% reduction).
- AP: Arm 2: 57.13 s → 4.54 s (92.1% reduction).
- AP: Arm 4: 90.59 s → 15.82 s (82.5% reduction).
- AP: Arm 5: 51.06 s → 2.45 s (95.2% reduction).

It is not possible to definitively confirm or refute Hypothesis H1, as the capacity calculation indicates that the TR may not always ensure a reduction in waiting time. The high volume of vehicles traveling on the roundabout can make it challenging for incoming vehicles to merge. The simulation confirmed very significant savings in delay time at all arms.

**H2:**  *The microsimulation of a roundabout provides more positive results than capacity calculations according to technical conditions.*

Assessment to H2: This hypothesis can certainly be confirmed, as the microsimulation confirmed significant improvements in the operational characteristics of the intersection, whereas the capacity calculation results in a slight improvement in the overall assessment of the intersection from level F to D or E, which is not a significant improvement.

**H3:**  *The queue length expressed in vehicles is reduced by the TR, but not on all entries compared to the SLR.*

**Figure 17:**
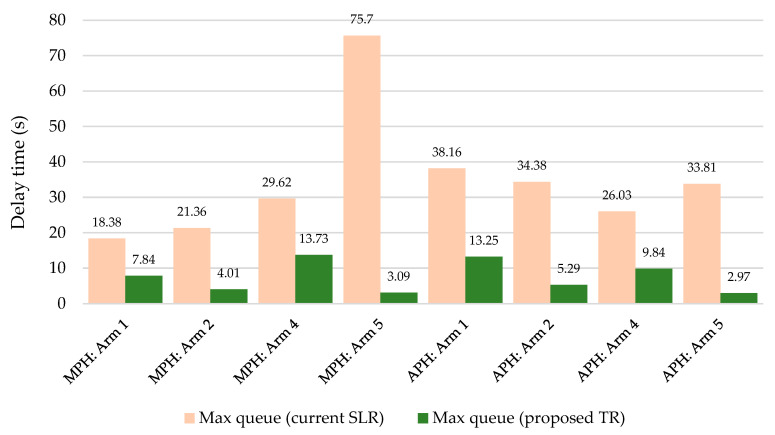
Maximum queue reduction according to microsimulation—TR compared to SLR. Source: authors.

Assessment to H3: This hypothesis can be rejected based on the results of the microsimulation, as the reduction in queue length can occur at all entries, as shown in Figure 17.

The results of the microsimulation for the additional TR highlight its operational efficiency under current traffic conditions. Table 10 summarizes the key performance indicators, including average delay time, LOS and vehicle count for MPH.

The total vehicle flow reached 2420.7 vehicles during MPH, with Entrance 4 handling the largest share of traffic at 1068.9 vehicles. Despite the varying traffic loads, the TR demonstrated consistent performance, with an average waiting time of 13.1 s across all entries. These findings further reinforce the versatility and reliability of TRs in managing diverse traffic demands. The results of the microsimulation for APH are presented in Table 11. The analysis once again demonstrates consistent operational performance at all entrances. The average delay time was 13.42 s which corresponds to a B-level LOS.

Shortest delay time was achieved at entrance 2 corresponding to 3.7 s while remaining entrances have delay time above 10 s corresponding to LOS level B. The APH managed to service larger number of vehicles 2432 while MPH managed to service 2421.

## 4. Discussion

Of course, the case study showed that the TR is not a universally suitable solution for all cases. The next step of the research was to examine and generalize the principles that apply to the load of a TR. With such an approach, it is possible to preliminarily determine the locations where a TR is appropriate.

For this purpose, an automatic capacity calculation algorithm was developed, with simulated traffic load of a TR in a standard configuration, i.e., with the following parameters:

- Main entries No. 2 and 4 have an entry type of 2/1 (two lanes at the entry, one circulating lane in the roundabout, complemented by a starting turbo lane).
- Main entries No. 1 and 3 have an entry type of 1/2 (one lane at the entry and two circulating lanes in the roundabout).
- Exit radius: 15 m.
- Even distribution of vehicles between the left and right lanes.

TR was analyzed using passenger car units (PCU) under five different scenarios, each reflecting a different share of main-direction traffic relative to the overall demand. For example, in scenario S70, 70% of PCUs traveled through the main direction—35% from arm 4 to arm 2 and 35% from arm 2 to arm 4. An additional 15% of traffic was assigned to right and left turns from the main directions, while the final 15% was distributed among all movements originating from arms 1 and 3.

**Figure 18:**
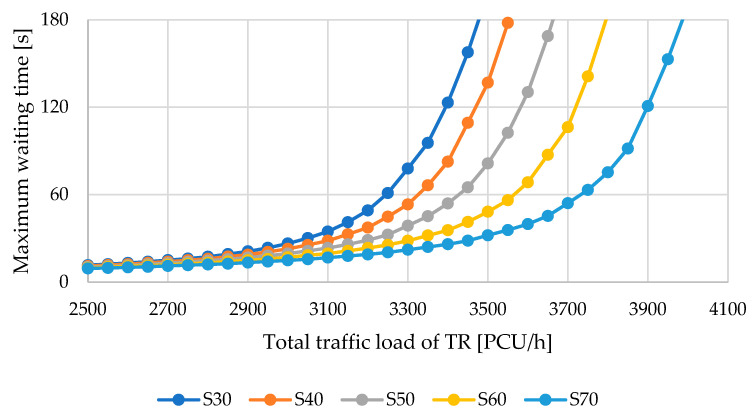
Development of maximum waiting time depending on various traffic load scenarios. Source: authors.

Figure 18 presents the evolution of the maximum waiting time, irrespective of the specific arm or lane where it occurred. Loads of up to 2500 PCU/hour are excluded from the figure since the roundabout can accommodate such volumes with waiting times below 10 s, corresponding to the highest level of service (LOS) grade “A,” regardless of traffic distribution. With increasing demand, however, the way traffic is allocated across directions becomes a critical factor. The most significant strain on the roundabout is naturally associated with left-turn movements and with flows entering from the secondary single-lane arms (1 and 3).

As can be seen from Figure 18, the intersection can manage 3500 PCU/h with an acceptable waiting time only in scenario S70, which represents a significant load in the main direction (70%), passing through the intersection as a four-lane road. In scenario S70, the maximum waiting time would be 32.1 s. In scenario S60, the maximum waiting time would be 48.3 s, and in the remaining cases, the waiting time would exceed one minute, with the intersection capacity being exceeded (negative capacity reserve).

**Figure 19:**
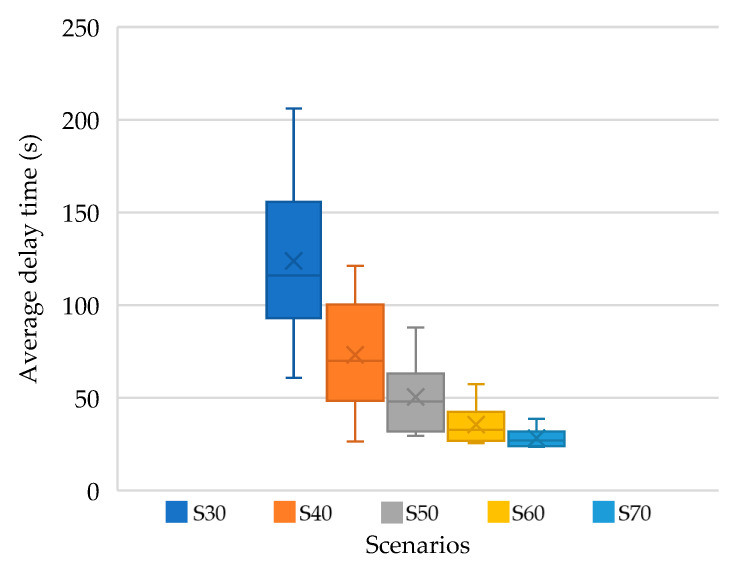
Development of average delay time from simulations depending on various traffic load scenarios. Source: authors.

Despite these pessimistic assumptions, all five scenarios with a load of 3500 PCU/h were evaluated by means of microsimulation. In the Aimsun Next 22.0.1.0 software, a TR was created on the drawing background, with the load defined by a passenger car matrix (since the capacity calculation is based on PCUs). Each variant was simulated with 25 replications, with a calculated average. Within microsimulation, the travel time was recorded and compared with the theoretical travel time on free-flow infrastructure. In this way, the delay time in seconds was obtained for each entry arm. The following figure (Figure 19) shows the information on the average delay time, whereas in the capacity calculation, the maximum value was always selected, regardless of which entry into the intersection it was measured at.

As can be seen from the figure, the intersection can indeed handle a load of 3500 PCU/h in scenarios S70 and S60, with a delay time below 60 s. In the case of negatively distributed loads in scenarios S50, S40, and S30, the intersection is unable to carry large traffic flows from the side directions, its capacity fails, and this results in a higher average waiting time with an increasing variance.

The calculated waiting time at an intersection based on capacity formulas may differ from the delay time obtained from a microsimulation model due to several reasons. Capacity calculations rely on simplified formulas, coefficients, and assumptions (e.g., average vehicle behavior, uniform traffic flow, constant arrival rates). These methods provide an exact numerical output but do not capture the stochastic and dynamic nature of real traffic. In microsimulation, vehicle movements are modeled individually, considering several parameters (for example, acceleration, deceleration, car-following, lane-changing behavior). This results in fluctuating waiting times and higher variability. Microsimulation also considers upstream and downstream interactions, as well as random disturbances, which cannot be fully reflected in static capacity equations.

Similarities can be found between this study and the research conducted by Leonardi and Distefano (2023) [86], who also analyzed the performance of turbo roundabouts using microsimulation techniques. Both studies aim to evaluate the operational efficiency and safety benefits of the turbo roundabout configuration compared to conventional layouts. In both cases, simulation-based performance indicators such as delay times and queue lengths were used to assess traffic flow quality under different demand conditions. Furthermore, both studies emphasize the importance of correct lane assignment and geometric design in ensuring the proper functioning of turbo roundabouts. The present research extends these findings by applying the methodology to a different spatial and regulatory context, providing additional evidence of the applicability of turbo roundabouts under Central European conditions.

However, this study has several limitations that may be subject to discussion. Firstly, the human factor was not considered. For instance, in [87], the authors note that drivers can make more than one hundred errors on a TR within an hour. Microsimulation and capacity calculations do not account for such deviations. Driver errors could not only increase waiting times, but in extreme cases could also cause an accident [88].

Further simplifications in the microsimulation include the use of only three vehicle categories (truck, bus, car) and the neglect of the minor impact of pedestrians and cyclists on the traffic flow.

Additionally, traffic safety was not assessed, although this would be difficult to evaluate within the scope of the design. In this regard, the authors in [1] state that the safety levels of standard roundabouts and TRs are approximately comparable.

It is clear that building small roundabouts outside urban areas is complicated and challenging. Traffic intensity can increase over several years, and a small roundabout can become an obstacle to smooth traffic flow. Better traffic throughput and fewer traffic jams can be achieved by changing the organization of traffic from a small roundabout to a TR, which is also applicable in this case. However, the study shows, similar to [89], that the quality of movement in a TR depends not only on the geometric arrangement but also on the distribution of load on individual entries and the direction of traffic flows.

Well-designed TRs can improve traffic operations compared to signal-controlled intersections, particularly under low to medium traffic demand [90]. Over the past 15 years, TRs have significantly advanced in geometry, capacity [91,92], safety, lifecycle efficiency, and environmental sustainability [93,94].

Modern advancements in vehicle performance, driven by innovations in the automotive industry, necessitate a broader examination of TRs across multiple dimensions. For the future of mobility, transportation engineers must understand how the increasing adoption of smart transportation technologies will impact road performance, specifically capacity, delays, and levels of service [95].

One key challenge is integrating autonomous vehicles and their navigation at roadways and intersections. This is crucial for future traffic control, policy-making, and highway design. The introduction of connected and autonomous vehicles (CAVs) is expected to significantly impact traffic safety and performance. Advances in vehicle-to-vehicle (V2V) and vehicle-to-infrastructure (V2I) communication technologies provide autonomous vehicles with new opportunities for traffic management [8,96].

Two-way communication can enhance the exchange of information, enabling greater collaboration between vehicles and road infrastructure. This technological evolution is anticipated to transform traffic systems over time. Connected and automated vehicles will likely improve traffic efficiency and safety, especially at urban intersections [97].

This study contributes to both traffic engineering practice and sensor-based transport research by combining field sensor measurements with analytical and simulation methods to evaluate the performance of TR.

Field data from radar and video sensors were used to calibrate and validate the microsimulation model, supporting robust estimates of delay, queue length, and saturation under real demand patterns. The derived capacity thresholds and lane-assignment recommendations provide actionable guidance for practitioners. l

## 5. Conclusions

Roundabouts are among the most effective types of intersections for managing traffic flow. Traditional SLRs have proven successful, particularly in areas with low to moderate traffic volumes. However, as traffic levels increase, these roundabouts can become congested, leading to more frequent accidents due to vehicle path crossings. TRs, which require drivers to select their lane in advance based on their intended exit, offer a solution that addresses some of the key drawbacks of conventional roundabouts.

This article examined the benefits and limitations of converting a traditional SLR into a TR. It compares capacity, safety, delays, and implementation costs, drawing on findings from traffic simulations and empirical studies. The analysis suggests that TRs perform better in high-traffic areas, enhancing safety and traffic flow. However, their implementation may involve higher costs and greater technical complexity.

From a scientific perspective, the article demonstrated that despite unsatisfactory results from the capacity assessment (with the overall evaluation of the roundabout at level E often considered unacceptable), the roundabout can achieve a high quality of traffic flow when assessed through microsimulation. The capacity assessment would not be improved even with the introduction of a separate bypass lane, as only about 10% of entering vehicles need to turn right.

According to the research presented in this paper, the threshold capacity of a TR is 3200 PCU/h, at which the roundabout in its standard configuration can handle the load under any traffic flow distribution. At higher volumes, it should be noted that a TR is suitable only when a high proportion of traffic flows in the main direction (at least 60%). For traffic volumes exceeding 3600 passenger cars per hour (PCU/h), even with a 70% share in the main direction, the roundabout is practically operating at full capacity.

The influence of pedestrians and cyclists must also be considered, as they significantly affect the continuity of traffic flow at the roundabout exits, especially under higher traffic intensities.

## Figures and Tables

**Table 1:** Traffic load matrix of the intersection for MPH.

Arm	1	2	3	4	5	Total
1	–	153	10	161	196	519
2	200	–	22	150	497	868
3	14	15	–	8	6	42
4	253	241	5	–	55	554
5	228	481	17	43	–	768
Total	694	888	54	362	753	2750

**Table 2:** Traffic load matrix of the intersection for APH.

Arm	1	2	3	4	5	Total
1	–	95	5	244	356	699
2	163	–	5	143	460	770
3	9	14	–	11	17	51
4	228	107	0	–	43	378
5	260	484	5	49	–	798
Total	660	699	14	446	876	2694

**Table 3:** Traffic quality levels and their brief characteristics.

LOS	Characteristic of the Waiting Time	Average Waiting Time w (s)
A	The waiting time is very short	≤10
B	Short waiting time without creating queues	≤20
C	Acceptable waiting time and rarely short queues	≤30
D	Steady state with high time losses	≤45
E	Unstable state	>45
F	Capacity exceeded	--- ^1^

^1^ Degree F is only achieved if the degree of saturation is greater than 1.

**Table 4:** Comparison of the results of the capacity assessment and the microsimulation.

Arm/Peak Hour	Average Queue Length—Traffic Survey (veh)	Average Queue Length—Simulation (veh)	Percentage Difference (%)
Arm 1—MP	5.06	5.35	+5.73
Arm 2—MP	16.89	17.59	+4.14
Arm 4—MP	16.81	17.11	+1.78
Arm 1—AP	9.51	9.41	−1.05
Arm 2—AP	28.10	27.60	−1.78
Arm 4—AP	5.33	5.53	+3.75

**Table 5:** Traffic growth coefficients according to TP 070.

Road Category	Vehicles	2023 ^1^	2033	2043
2nd class	light vehicles	1.000	1.085	1.140
	heavy vehicles	1.000	1.085	1.146
3rd class	light vehicles	1.000	1.073	1.166
	heavy vehicles	1.000	1.084	1.138

^1^ All values are converted to the current state—year 2023.

**Table 6:** Results of capacity assessment for SLR according to TP 102.

Year	Variable	Arm 1	Arm 2	Arm 3	Arm 4	Arm 5
2023 MPH	*w_i_*	22.1	63.6	17.7	119.3	206.8
2023 MPH	LOS	C	E	B	F	F
2033 MPH	*w_i_*	49.2	205.8	26.2	448.9	516.1
2033 MPH	LOS	E	F	C	F	F
2043 MPH	*w_i_*	161.7	364.2	40.3	846.4	801.7
2043 MPH	LOS	F	F	D	F	F
2023 APH	*w_i_*	34.6	187.2	35.4	33.4	37.3
2023 APH	LOS	D	F	D	D	D
2033 APH	*w_i_*	116.1	465.0	105.9	116.3	126.6
2033 APH	LOS	F	F	E	E	F
2043 APH	*w_i_*	313.4	732.9	1289.4	449.7	278.5
2043 APH	LOS	F	F	F	F	F

**Table 7:** Results of capacity assessment for proposed TR according to TP 100. Source: authors.

Year	Variable ^1^	Arm 1	Arm 2L	Arm 2R	Arm 4	Arm 5L	Arm 5R
2023 MPH	*w_i_*	16.0	6.4	7.1	32.1	9.8	11.2
2023 MPH	LOS	B	A	A	D	A	B
2033 MPH	*w_i_*	19.6	6.8	7.5	49.4	11.2	13.0
2033 MPH	LOS	B	A	A	E	B	B
2043 MPH	*w_i_*	23.1	7.2	8.0	73.7	12.8	15.2
2043 MPH	LOS	C	A	A	E	B	B
2023 APH	*w_i_*	11.8	10.2	11.7	53.6	6.8	7.6
2023 APH	LOS	B	B	B	E	A	A
2033 APH	*w_i_*	13.4	11.5	13.5	101.4	7.3	8.1
2033 APH	LOS	B	B	B	E	A	A
2043 APH	*w_i_*	14.8	13.4	16.1	176.8	7.8	8.8
2043 APH	LOS	B	B	B	F	A	A

^1^ Arm 3 was led outside the intersection in the proposal.

**Table 8:** Results of microsimulation (64 replications) for current SLR and proposed TR (MP).

Type	Variable	Arm 1	Arm 2	Arm 3	Arm 4	Arm 5
SLR	Delay time (s)	30.79	18.92	16.01	273.95	218.40
TR	Delay time (s)	7.02	2.55	–	17.65	2.76
SLR	Stop time (s)	26.46	12.90	15.89	271.02	202.02
TR	Stop time (s)	4.72	1.89	–	14.53	1.41
SLR	Max queue (veh)	18.38	21.36	2.48	29.62	75.70
TR	Max queue (veh)	7.84	4.01	–	13.73	3.09

**Table 9:** Results of microsimulation (64 replications) for current SLR and proposed TR (AP).

Type	Variable	Arm 1	Arm 2	Arm 3	Arm 4	Arm 5
SLR	Delay time (s)	72.96	57.13	28.97	90.59	51.06
TR	Delay time (s)	12.63	4.54	–	15.82	2.45
SLR	Stop time (s)	62.67	47.18	29.04	87.75	41.08
TR	Stop time (s)	8.95	3.73	–	13.48	1.11
SLR	Max queue (veh)	38.16	34.38	3.16	26.03	33.81
TR	Max queue (veh)	13.25	5.29	–	9.84	2.97

**Table 10:** Summary of microsimulation results for additional TR, MPH.

Variable	Entrance 1	Entrance 2	Entrance 3	Entrance 4	Average	∑	LOS
*w_i_*	17.93	3.3	12.63	18.57	13.1075	-	B
LOS	B	A	B	B	-	-	
Vehicle count	108.3	824	419.5	1068.9	-	2420.7	

**Table 11:** Summary of microsimulation results for additional TR, APH.

Variable	Entrance 1	Entrance 2	Entrance 3	Entrance 4	Average	∑	LOS
*w_i_*	19.45	3.7	13.7	16.81	13.415	-	B
LOS	B	A	B	B	-	-	
Vehicle count	102.97	848.35	422.00	1058.65	-	24,319.7	

## Data Availability

All used data are available on request from the author.
